# Editorial: Multidisciplinary approaches to diagnosis and management of traumatic encephalopathy syndrome (TES)

**DOI:** 10.3389/fneur.2023.1254333

**Published:** 2023-08-29

**Authors:** Roy G. Beran

**Affiliations:** ^1^University of New South Wales, Kensington, NSW, Australia; ^2^Griffith University, Southport, QLD, Australia; ^3^Western Sydney University, Sydney, NSW, Australia; ^4^Sechenov First Moscow State University, Moscow, Russia

**Keywords:** multidisciplinary, traumatic brain injury, chronic traumatic encephalopathy syndrome, traumatic encephalopathy syndrome, potential intervention, basic pathophysiology

Traumatic encephalopathy syndrome (TES) and chronic traumatic encephalopathy (CTE), are major issues when reviewing concussion and traumatic brain injury (TBI) and have emerged as serious health considerations in the 21st century. Their significance was acknowledged and their Importance assumed center stage as one of the main themes in the 26th World Congress for Medical Law, hosted by the World Association for Medical Law, in December 2022. It is absolutely appropriate that this topic, being a multidisciplinary approach to the diagnosis and management of TES, be the subject of a Research Topic of Frontiers in Neurology.

This Research Topic covers so many aspects of TES and CTE, from: “cause and effect,” with relevance to recurrent head impacts (RHI; applying the Bradford Hill Criteria) (Nowinski et al.); evaluation of impact and prognosis (looking at the predictive value of various shock indices combined with the Glasgow Coma Scale) (Lin et al.); discussion of the underlying pathophysiology, with specific analysis of the direct effects of inflammation and the kynurenine pathway, leading to consideration of potential therapeutic intervention (Dehhaghi et al.); the need to be able to offer an antemortem diagnosis of TES, accepting that the diagnosis of CTE is limited to a postmortem examination, combined with relevance to possible antemortem intervention in a multidisciplinary team (Kim and Beran); and the value of determining correlation of various coefficient determinations, based on intracranial pressure wave amplitude and resistance measurements for cerebro-spinal fluid (CSF) outflow, to predict recovery following ventriculo-peritoneal shunting, as may be required in posttraumatic hydrocephalus (Zhang et al.).

Nowinski et al. accepted that recognition of CTE dates back to the early 20th century and that it has a relationship to the effects of RHI, especially those associated with contact sports. They identified the need to confirm that this is a “cause and effect” relationship and applied the 9 points, as proposed by Sir Austin Bradford Hill, in 1965. They argued that this offered the best available evidence to underwrite a “cause and effect” relationship between CTE and RHI and they raised the legal medicine question of having children who are below the age of consent being exposed to the effects of contact sports. There is a basic premise that parents will act in the best interests of the child but only time will tell if allowing children to be exposed to RHI, as is the case in contact sport, will be the subject of litigation between children and their parents, once those children come of age and reach adulthood. It is a somewhat different argument for adults because it is proffered that those adults who willing engage in such contact sports do so knowingly, accepting the risk of traumatic brain injury (TBI), and the risk is no longer material to their decision making (1).

Evidence from a 10 year, single center analysis of the predictive value of shock index, modified shock index, age adjusted shock index and reverse shock index multiplied by the Glasgow Coma Scale (GCS) was used to determine mortality risk following TBI in more than 1,700 TBI patients (Lin et al.). The authors looked at various factors, such as: age; injury mechanism; concurrent illnesses; severity of TBI; and the severity of the injury. They concluded that the revreve shock index, multiplied by the GCS provided the best predictive indicator, especially in moderate to severe TBI or with isolated head injury, to more accurately predict mortality in TBI patients.

Dehhaghi et al. looked at the potential pathophysiological mechanisms of brain damage which are associated with mild TBI, as occurs in 80−90% of the 10 million who experience TBI on an annual basis. They provided the hypothesis that the TBI initiates a neurochemical cascade with inflammation, excitotoxicity and consequences of reactive oxygen species (ROS). They identified primary TBI, being the direct effects of the trauma on brain tissue, and secondary damage, due to biomolecular changes and pathophysiological consequences, resulting in cerebral oedema, hemorrhage, infection and ischemia. They discussed neurochemical changes, following the initial TBI, resulting in release of excitatory amino acids (such as glutamate), generating ROS and nitric oxide causing brain cell injury. The process included an influx of calcium into brain cells with excitatory amino acids which could then promote ROS, within the neurones, resulting in nitric acid release causing oxidative stress, lipid peroxidation and release of excitatory amino acids with glutamate increasing the influx of calcium into the neurones post TBI. On this basis, they argued in favor of receptor antagonists, such as N-methyl-D-aspartic acid (NMDA) and amino-3-hydroxy-5 methyl-4-isoxazole proprionic acid (AMPA) receptor antagonists, playing an active role in modifying and improving the prognosis following TBI, possibly by reducing the subsequent inflammation.

In their analysis, Dehhaghi et al. suggested that TBI activated the inflammatory pathways recruiting microglia and peripheral neutrophils, resulting in the infiltration of macrophages and leukocytes. This led to the release of cytokines and chemokines and activation of the kynurenine pathway, resulting in the production of quinolinic acid, kynurenine and 3-hydroxykinurenine all of which are known to be associated with such conditions as Alzheimer's disease or Amyotrophic Lateral Sclerosis (ALS). These conditions are also known to be associated with TES and CTE. On this foundation of TBI induced post TBI neurotoxicity, they argued in favor of using an NMDA receptor antagonist, such as ketamine to reduce the inflammatory effects and subsequent brain damage. This concept opens a whole new window for consideration of possible prevention of the consequences of TBI. Fycompa (perampanel) is an approved adjunctive antiseizure medication that works as a non-competitive AMPA receptor antagonist (2) and may, on the basis of the hypothesis suggested by Dehhaghi et al., have a therapeutic role in the prevention of the effect of mild TBI and the subsequent inflammation. There are no trials of this approach to TBI but the concept seems fertile. Other NMDA receptor antagonists include amantadine which was first developed as an influenza remedy and subsequently is used to treat Parkinson's disease (3), another condition which is increased in those with TES and CTE. What this suggests is that there is a large gray area in the pharmaceutical management of TES and CTE that remains to be explored.

When considering intervention for TES or CTE there exists a major hurdle in that the antemortem diagnosis of CTE remains controversial. Kim and Beran point out the need for there to be a wider acceptance of the concept of TES representing an antemortem diagnosis of CTE to allow intervention and appropriate management. They too raise the legal medicine considerations of the need for clinicians to offer a correct diagnosis following which appropriate management can ensue. Without an accepted diagnosis, any intervention is largely speculative and may leave the clinician vulnerable. Even the earlier referenced article, by Nowinski et al., seems to have accepted CTE as an antemortem diagnosis which is technically incorrect but, as Kim and Beran point out, the absolute diagnosis of Parkinson's disease or Alzheimer's disease, both of which seem to be more prevalent in those who had RHI and TBI, remains a postmortem diagnosis but clinicians have accepted the syndromic equivalent and the antemortem introduction of remedies based on the presumed antemortem label. In a separate article, Kim and Beran have also raised the question of the use of other newer treatments for Alzheimer's disease in the potential armamentarium for managing TES and CTE (4), acknowledging the increase of dementia in those with TES and CTE.

The role of intervention for TES and CTE extends far beyond the pharmacological means of modifying neural receptors and must include a vast army of therapists whose role extends beyond simply treating the index patients. Kim and Beran highlight the need to include the family in the therapeutic model, to help manage the potential disruptive behavior. There is need to treat the various auxiliary, concomitant conditions that appear increased within the context of TES/CTE, such as the increase in Alzheimer's disease, ALS or Parkinson's disease that has already been raised within this editorial. There may be need for occupational therapy, rehabilitation, psychotherapy and the involvement of a range of allied health professionals without whom the patient and his/her family are left to fend for themselves.

While TES/CTE are often associated with premature death, Zhang et al. have examined the value of tools to predict prognosis in those with TBI and hydrocephalus requiring ventriculo-peritoneal shunt. In their study of 70 patients, they confirmed that the correlation coefficient between the intracranial pressure wave amplitude and the mean intracranial pressure level and resistance to CSF outflow can predict desirable recovery level.

While this Research Topic includes a limited number of papers, the contribution of those who have submitted their work is a forceful overview of the current status of the understanding of TES/CTE and offers considerable food for thought. Those involved have made what is a complex topic more understandable for those who are on the periphery of TES/CTE. The limited scope of this Research Topic belies the vast extent of the content of material which, through the involvement of people from different backgrounds and levels of expertise, has provide a valuable overview of multidisciplinary approaches to the diagnosis and management of TES.

## Author contributions

RB: Conceptualization, Writing—original draft, Writing—review and editing, Formal analysis, Investigation, Methodology, Project administration, Resources, Supervision, validation.
